# Performance evaluation of mainstream large language models in autoimmune hepatitis patient education: a comparative study of readability, quality, and reliability

**DOI:** 10.3389/fpubh.2026.1805848

**Published:** 2026-03-20

**Authors:** Hanlu Li, Yuru Lin, Lin Lv

**Affiliations:** 1 Department of Infectious Diseases, Beibei Affiliated Hospital of Chongqing Medical University, Chongqing, China; 2 Department of Gastroenterology, The Second Affiliated Hospital of Chongqing Medical University, Chongqing, China

**Keywords:** autoimmune hepatitis, health information quality, large language models, patient education, readability

## Abstract

**Background:**

Autoimmune hepatitis (AIH) is a chronic immune-mediated liver disease that requires long-term management, in which effective patient education plays a critical role. With the rapid development of large language models (LLMs), AI-generated health information is increasingly accessed by patients; however, the readability, quality, and educational suitability of LLM-generated AIH-related content remain insufficiently evaluated.

**Methods:**

Five widely used LLMs—ChatGPT, Doubao, DeepSeek, Wenxin Yiyan, and Tongyi Qianwen—were assessed based on their responses to 20 frequently asked AIH patient education questions covering five thematic categories. Text readability was evaluated using multiple indices, including the Automated Readability Index, Flesch Reading Ease Score, Gunning Fog Index, Flesch–Kincaid Grade Level, Coleman–Liau Index, SMOG, and Linsear Write formula. Information quality and educational suitability were assessed using the Global Quality Score (GQS) and the Chinese version of the Patient Education Materials Assessment Tool (C-PEMAT). Clinical Intent Alignment (CIA) was used to evaluate the coverage of guideline-defined medical key points based on the 2025 EASL Clinical Practice Guidelines. Inter-rater reliability was analyzed using Cohen’s kappa, and comparative and correlation analyses were performed.

**Results:**

Significant differences were observed among the LLMs in readability, information quality, and educational suitability (all *p* < 0.05). ChatGPT achieved the highest GQS and C-PEMAT scores, followed by Doubao and DeepSeek, whereas Wenxin Yiyan and Tongyi Qianwen showed lower performance and greater variability. CIA analysis indicated comparable coverage of guideline-defined clinical intent across models. Readability varied significantly across content themes, with texts related to disease mechanisms and diagnostic processes exhibiting higher linguistic complexity. Correlation analysis demonstrated moderate associations between GQS and grade-level readability indices, whereas C-PEMAT and CIA showed weak correlations with traditional readability metrics.

**Conclusion:**

Substantial variability exists among LLMs in generating AIH patient education materials. Model selection critically influences information quality and educational suitability, whereas content theme primarily affects linguistic complexity. Although most models produced moderate-to-good quality information, relatively high readability levels suggest that further simplification may be needed for general patient populations. A multidimensional evaluation framework integrating readability, quality, educational suitability, and clinical intent alignment is essential for the responsible use of LLMs in AIH patient education.

## Introduction

Autoimmune hepatitis (AIH) is a chronic immune-mediated inflammatory liver disease characterized histologically by hepatocellular necrosis and portal inflammatory cell infiltration. Clinically, AIH is commonly associated with elevated serum transaminase levels, the presence of autoantibodies, and increased immunoglobulin G (IgG) concentrations. The onset of AIH is often insidious, and its clinical presentation is highly heterogeneous, ranging from asymptomatic liver enzyme abnormalities to acute or even fulminant liver failure. Without timely diagnosis and standardized treatment, a proportion of patients may progress to liver cirrhosis or end-stage liver disease, resulting in impaired long-term prognosis and reduced quality of life (1–3). In recent years, the incidence of AIH has shown an increasing trend across multiple countries, with a higher prevalence observed among women and middle-aged to older adults (4). As the number of affected individuals continues to rise, challenges related to the long-term management of AIH and the delivery of effective patient education have become increasingly prominent (5). Although the precise pathogenesis of AIH has not been fully elucidated, current evidence suggests that its development is closely associated with genetic susceptibility, loss of immune tolerance, environmental triggers, and dysregulation of the gut–liver axis (6, 7). Immunosuppressive therapy is effective in controlling disease activity in most patients; however, AIH typically requires prolonged or lifelong treatment and follow-up, with relatively high relapse rates. In addition, adverse effects related to corticosteroids and immunosuppressive agents remain a significant clinical concern (8, 9). Previous studies have demonstrated that patients’ understanding of their disease, treatment objectives, and the balance between therapeutic benefits and risks has a direct impact on treatment adherence, follow-up behaviors, and overall clinical outcomes (10, 11). Therefore, providing accurate, evidence-based, and comprehensible health education materials is a critical component of comprehensive chronic disease management for patients with AIH. With the widespread availability of the internet and the rapid expansion of online question-and-answer platforms and social media, patients’ access to medical information has gradually shifted from traditional face-to-face physician–patient communication toward digital and self-directed information-seeking approaches (12). However, the quality of online medical information is highly variable. Incomplete, overly simplified, or non–evidence-based content may lead to disease misinterpretation, delayed treatment, or unnecessary anxiety among patients (13, 14). Achieving an appropriate balance between information accessibility and scientific rigor has thus emerged as a major challenge in contemporary patient education.

In recent years, advances in artificial intelligence (AI), particularly the development of large language models (LLMs), have introduced new tools for medical knowledge dissemination and patient education (15). By learning from large-scale textual data, these models are capable of generating structured and linguistically coherent responses through natural language interactions. International models such as ChatGPT have demonstrated potential applications in medical question answering, patient consultation, and medical education (16). Meanwhile, several general-purpose LLMs developed in China have been rapidly adopted across Chinese digital platforms, creating a unique Chinese-language ecosystem for AI-assisted health information seeking. Nevertheless, the use of LLMs in medical health education remains subject to substantial uncertainty, including concerns regarding the accuracy of medical information, response consistency, control of linguistic complexity, and alignment with patients’ health literacy levels (17, 18). Recent studies have begun to explore the use of LLMs in autoimmune liver diseases, including autoimmune hepatitis. For instance, a recent investigation assessed the reliability of ChatGPT-generated responses for AIH-related clinical questions and reported generally acceptable accuracy but variability in informational completeness across domains (19). Another comparative study evaluating multiple chatbot systems in autoimmune liver disease similarly found that although LLMs may provide broadly correct information, differences remain in consistency, clinical depth, and guideline alignment (20). However, most existing studies have focused primarily on response accuracy or reliability, whereas comprehensive evaluations integrating readability, information quality, and patient education suitability remain limited, particularly within Chinese-language health information environments. These limitations may influence patients’ comprehension and trust in AI-generated information, thereby constraining the real-world value of LLMs in patient education. Moreover, potential differences among LLMs in text quality, readability, and educational suitability warrant systematic comparison and quantitative evaluation.

To date, systematic assessments of LLM-generated patient education content in the context of AIH—a chronic autoimmune liver disease requiring long-term management—remain limited. In particular, comprehensive comparative analyses incorporating multiple dimensions such as readability, overall information quality, and patient education suitability are lacking. Therefore, the present study evaluated patient education texts generated by five widely used LLMs—ChatGPT, Doubao, DeepSeek, Wenxin Yiyan, and Tongyi Qianwen—based on common AIH-related patient education questions. These models represent a combination of internationally used and widely accessible Chinese-language LLM platforms, enabling assessment of AI-generated health information within the Chinese-language digital health information environment. This model selection strategy was intended to reflect realistic access patterns in Chinese-language patient information settings, where domestically developed LLMs are more commonly available and used than other leading international platforms such as Claude or Gemini. The generated content was systematically evaluated across multiple dimensions, including readability, overall information quality using the Global Quality Score (GQS), patient education suitability using the Chinese version of the Patient Education Materials Assessment Tool (C-PEMAT), and guideline-aligned clinical content coverage assessed through Clinical Intent Alignment (CIA). By integrating these complementary evaluation metrics, this study aims to provide a comprehensive assessment of the reliability and educational suitability of LLM-generated AIH information in Chinese-language patient education contexts.

## Materials and methods

### Ethical considerations

All data used in this study were derived exclusively from text generated by large language models and did not involve any human participants or animal experiments. No personal identifiable information, biological specimens, or clinical data from real individuals were collected, used, or analyzed. Therefore, this study complied with relevant academic ethical standards and did not require approval from an institutional ethics committee.

### Research procedure

On December 15, 2025, two clinical experts jointly developed and finalized 20 frequently asked questions related to autoimmune hepatitis (AIH) (Table 1). The question framework was informed by the core domains outlined in the 2025 EASL Clinical Practice Guidelines and refined through expert discussion to reflect common clinical consultation topics. These questions were categorized into five thematic domains based on content attributes: disease etiology and mechanisms, clinical manifestations, diagnostic evaluation, treatment and management, and prognosis and follow-up.

**Table 1:** Issue list.

**Issue list**
Etiopathogenesis dimension
1. What is the core immune abnormality causing autoimmune hepatitis?
2. What is the role of genetic factors in the pathogenesis of autoimmune hepatitis?
3. Can infections or drugs induce autoimmune hepatitis?
4. What is the association between autoantibody production and hepatocyte damage?
Clinical manifestations dimension
5. What are the characteristics of common symptoms such as fatigue and jaundice in autoimmune hepatitis?
6. Can some patients present with only elevated liver enzymes as the initial manifestation?
7. What complications may occur in advanced autoimmune hepatitis?
8. Is autoimmune hepatitis accompanied by extrahepatic autoimmune diseases?
Diagnostic examinations dimension
9. Which characteristic autoantibodies need to be detected for autoimmune hepatitis?
10. What is the key value of liver biopsy in confirming the diagnosis?
11. How to exclude similar diseases such as viral hepatitis during diagnosis?
12. What are the reference ranges for the elevation of serum transaminases and immunoglobulins?
Treatment and management dimension
13. What are the first-line therapeutic drugs for autoimmune hepatitis?
14. How to control the dosage adjustment and course of hormone therapy?
15. What second-line treatment options are available for refractory cases?
16. Which key indicators need to be regularly monitored during treatment?
Prognosis and follow-up dimension
17. What is the prognosis of autoimmune hepatitis after standardized treatment?
18. What are the risk factors and early warning signs of disease recurrence?
19. What is the frequency of long-term follow-up and the core examination items?
20. What should patients pay attention to in daily diet and lifestyle?

The finalized questions were then independently input into five widely accessible large language models that were publicly available at the time of the study, including ChatGPT (GPT-5.1^1^), Doubao^2^, DeepSeek^3^, Wenxin Yiyan^4^, and Tongyi Qianwen^5^. The responses generated by each model were systematically evaluated and compared across three dimensions: readability, reliability, and overall information quality. All questions were entered directly into the public web interfaces of the evaluated models using a zero-shot prompting strategy without additional role instructions or prompt engineering. Detailed information regarding the prompting strategy, model configuration, and example interaction is provided in Supplementary Table S1.

### Readability evaluation

All prompts and responses were conducted in Chinese to reflect real-world patient language use in our study context. For readability assessment, the generated Chinese responses were translated into English by two independent hepatologists with expertise in autoimmune hepatitis, and any discrepancies were resolved through consensus prior to analysis.

Currently, no widely adopted and internationally validated Chinese readability index directly comparable to standard English syllable-based formulas is available. Therefore, readability was assessed using the Readability Formulas online tool^6^.

The following indices were applied: Coleman–Liau Index (CLI), Linsear Write (LW), Automated Readability Index (ARI), Simple Measure of Gobbledygook (SMOG), Gunning Fog Index (GFOG), Flesch Reading Ease Score (FRES), and Flesch–Kincaid Grade Level (FKGL). These metrics estimate text difficulty based on linguistic features such as sentence length, word length, and syllable or character complexity. Most indices produce an estimated reading grade level indicating the education required to understand the text, whereas FRES provides an inverse measure in which higher scores indicate easier readability. The scores were calculated automatically using the online tool and interpreted as relative indicators of linguistic complexity rather than precise measures of native-language readability.

### Quality assessment

The reliability and quality of the AI-generated responses were evaluated using the Chinese version of the Patient Education Materials Assessment Tool for printable materials (C-PEMAT-P) and the Global Quality Score (GQS).

The C-PEMAT-P includes 24 items across two domains: understandability (16 items), assessing clarity, organization, and the use of lay language, and actionability (8 items), evaluating whether clear and practical recommendations are provided. Each item is scored as 0 (not met) or 1 (met), yielding a total score ranging from 0 to 24, with higher scores indicating better patient accessibility.

The Global Quality Score (GQS) is a five-point Likert scale used to evaluate overall information quality, ranging from 1 (poor quality) to 5 (excellent quality with comprehensive, well-structured, and highly useful information).

On December 25, 2025, all responses were independently evaluated using both instruments by two clinical experts with more than 3 years of relevant clinical experience. In cases of scoring discrepancies, a third expert was consulted to adjudicate differences and reach a consensus through discussion. Inter-rater reliability was quantified using Cohen’s kappa coefficient, with values interpreted as follows: *κ* > 0.75 indicating excellent agreement, 0.40 ≤ *κ* ≤ 0.75 indicating acceptable agreement, and κ < 0.40 indicating poor agreement. The results demonstrated that the Cohen’s kappa coefficients for both the C-PEMAT-P and GQS exceeded 0.75, indicating high inter-rater agreement and reliability. Importantly, the interquartile ranges (IQRs) reported in the results tables reflect variability across the 100 model-generated responses (20 questions × five LLMs) rather than disagreement between raters.

### Clinical intent alignment (CIA) evaluation

Clinical Intent Alignment (CIA) was used as an AI-specific evaluation metric to assess whether LLM-generated responses adequately covered guideline-defined clinical key points. The CIA scoring approach was inspired by rubric-based evaluation frameworks for medical dialogue systems proposed in recent LLM evaluation studies (21). For each question, key clinical elements were identified based on the 2025 EASL Clinical Practice Guidelines for autoimmune hepatitis. CIA scores were calculated as the proportion of key points addressed in each response, with values ranging from 0 to 1, where higher scores indicated greater alignment with core clinical intent.

### Statistical analysis

Continuous variables following a normal distribution were expressed as mean ± standard deviation (Mean ± SD), and comparisons among multiple groups were performed using one-way analysis of variance (ANOVA). For continuous variables that did not conform to a normal distribution, data were presented as medians with interquartile ranges, and group differences were assessed using the Kruskal–Wallis H test. All statistical tests were two-tailed, with a significance level set at *p* < 0.05. Statistical analyses were conducted using IBM SPSS Statistics version 25.0, and figures were generated using GraphPad Prism version 9.0. Because the primary aim of this study was exploratory comparison of performance across multiple LLMs rather than testing a single confirmatory hypothesis, no formal correction for multiple comparisons was applied, and the statistical results should therefore be interpreted as exploratory.

## Results

### Readability analysis

This study evaluated the effects of different large language models and health education themes on the readability, educational suitability, information quality, and clinical intent alignment of AIH patient education materials. At the model level, C-PEMAT, GQS, CIA, and multiple readability indices (ARI, FRES, GFOG, FKGL, CL, SMOG, and LW) were compared across five widely used LLMs (ChatGPT, DeepSeek, Doubao, Tongyi Qianwen, and Wenxin Yiyan). At the content level, these metrics were further analyzed across five AIH-related educational themes (etiology and mechanisms, clinical manifestations, diagnostic evaluation, treatment and management, and prognosis and follow-up).

With respect to patient education suitability (Table 2), ChatGPT-generated texts achieved the highest C-PEMAT scores (8.25 ± 1.21), followed by Doubao (8.00 ± 1.38) and Tongyi Qianwen (7.75 ± 0.85). DeepSeek showed intermediate performance (7.70 ± 0.86), whereas Wenxin Yiyan received the lowest scores (6.95 ± 1.23). The differences among models were statistically significant (*p* = 0.007). A similar trend was observed for overall quality as assessed by GQS, with ChatGPT demonstrating the highest scores (4.30 ± 0.47), followed by DeepSeek and Doubao (both 3.40 ± 0.50). Tongyi Qianwen and Wenxin Yiyan exhibited lower GQS scores, with highly significant inter-model differences (*p* < 0.001). These findings indicate that ChatGPT outperformed other models in terms of content structure, information organization, and overall quality of AIH patient education materials.

**Table 2:** Comparison of text quality and readability across different large language models.

Variables	Total (*n* = 100)	Deep seek (*n* = 20)	Doubao (*n* = 20)	GPT-5 (*n* = 20)	Tongyi Qianwen (*n* = 20)	Wenxin Yiyan (*n* = 20)	Statistic	*P*
C-PEMAT score, Mean ± SD	7.73 ± 1.19	7.70 ± 0.86	8.00 ± 1.38	8.25 ± 1.21	7.75 ± 0.85	6.95 ± 1.23	*F* = 3.75	0.007
GQS score, Mean ± SD	3.22 ± 0.89	3.40 ± 0.50	3.40 ± 0.50	4.30 ± 0.47	3.00 ± 0.46	2.00 ± 0.56	*F* = 55.24	<0.001
CIA, M (Q₁, Q₃)	0.70 (0.60, 0.80)	0.70 (0.60,0.80)	0.65 (0.60,0.72)	0.70 (0.67,0.80)	0.70 (0.57,0.80)	0.70 (0.57,0.80)	χ^2^ = 1.60#	0.809
ARI, M (Q₁, Q₃)	17.79 (15.95, 19.98)	16.68 (14.46,18.81)	20.05 (18.25,22.20)	19.72 (18.59,20.89)	17.34 (16.12,18.57)	15.05 (13.86,16.11)	χ^2^ = 46.56#	<0.001
FRES, M (Q₁, Q₃)	12.00 (0.00, 24.00)	12.00 (3.25,16.25)	5.00 (0.00,14.00)	0.00 (0.00,7.50)	14.00 (0.00,20.50)	27.50 (23.75,38.25)	χ^2^ = 34.44#	<0.001
GFOG, M (Q₁, Q₃)	17.80 (15.95, 19.22)	17.75 (17.10,19.20)	18.80 (17.70,20.12)	19.20 (17.78,20.72)	17.75 (16.38,18.70)	13.65 (12.78,15.88)	χ^2^ = 37.18#	<0.001
FKGL, M (Q₁, Q₃)	16.27 (14.08, 18.16)	15.17 (14.07,17.39)	17.79 (16.31,19.91)	18.31 (17.03,19.50)	15.68 (14.01,17.85)	13.97 (12.79,15.26)	χ^2^ = 35.72#	<0.001
CL, M (Q₁, Q₃)	18.89 (16.46, 20.49)	18.91 (17.03,20.20)	19.64 (17.17,21.65)	20.02 (18.96,21.68)	18.92 (17.57,20.39)	14.41 (13.04,16.26)	χ^2^ = 36.97#	<0.001
SMOG, M (Q₁, Q₃)	13.91 (12.12, 15.23)	12.91 (11.75,14.11)	15.34 (14.11,16.89)	15.23 (14.21,16.26)	13.12 (12.21,14.31)	11.92 (11.22,12.91)	χ^2^ = 44.20#	<0.001
LW, M (Q₁, Q₃)	54.00 (51.00, 58.00)	55.00 (52.00,58.00)	51.00 (47.50,53.25)	51.50 (50.00,55.25)	56.00 (53.00,58.00)	59.00 (56.75,61.25)	χ^2^ = 28.05#	<0.001

F: ANOVA, #: Kruskal-Wallis test. SD: standard deviation, M: Median, Q₁: 1st Quartile, Q₃: 3st Quartile.

Significant differences were also observed across all models for readability-related indices, including ARI, FRES, GFOG, FKGL, CL, SMOG, and LW (all *p* < 0.001). ChatGPT and Doubao exhibited higher values for ARI, GFOG, FKGL, CL, and SMOG, indicating relatively greater linguistic complexity and higher reading demands. In contrast, Wenxin Yiyan showed lower scores on these indices, suggesting a comparatively lower reading burden. However, an opposite pattern was observed for the LW index, with Wenxin Yiyan demonstrating the highest proportion of long words, while ChatGPT and Doubao showed relatively lower values.

Content-level analysis (Table 3) revealed significant differences across AIH health education themes in terms of patient education suitability and text readability. C-PEMAT scores differed significantly among content categories (*F* = 17.91, *p* < 0.001), with higher scores observed for treatment and management as well as prognosis and follow-up topics, and lower scores for disease etiology and pathogenesis. In contrast, overall quality assessed by GQS did not differ significantly across themes (*p* = 0.729). Regarding readability, significant differences among content categories were identified for FRES (*p* = 0.009), FKGL (*p* = 0.005), and SMOG (*p* = 0.026). Texts related to disease mechanisms and diagnostic evaluation required higher reading grade levels, indicating increased linguistic complexity. Other readability indices, including ARI, GFOG, CL, and LW, did not reach statistical significance (*p* > 0.05), although similar trends were observed.

**Table 3:** Comparison of text quality and readability across different health education content categories.

Variables	Total (*n* = 100)	Clinical manifestations dimension (*n* = 20)	Diagnostic examinations dimension (*n* = 20)	Etiopathogenesis dimension (*n* = 20)	Prognosis and follow-up dimension (*n* = 20)	Treatment and management dimension (*n* = 20)	Statistic	*P*
C-PEMAT score, Mean ± SD	7.73 ± 1.19	7.20 ± 0.83	7.35 ± 0.88	6.80 ± 1.11	8.55 ± 0.89	8.75 ± 0.85	*F* = 17.91	<0.001
GQS score, Mean ± SD	3.22 ± 0.89	3.45 ± 0.89	3.20 ± 1.06	3.20 ± 0.70	3.20 ± 0.83	3.05 ± 1.00	*F* = 0.51	0.729
CIA, M (Q₁, Q₃)	0.70 (0.60, 0.80)	0.70 (0.67,0.72)	0.65 (0.47,0.83)	0.80 (0.80,0.90)	0.70 (0.60,0.70)	0.60 (0.50,0.70)	χ^2^ = 17.9#	0.001
ARI, M (Q₁, Q₃)	17.79 (15.95, 19.98)	19.48 (16.80,20.97)	17.01 (14.47,20.11)	19.20 (17.50,20.17)	16.34 (15.66,18.06)	17.43 (16.50,18.65)	χ^2^ = 9.43#	0.051
FRES, M (Q₁, Q₃)	12.00 (0.00, 24.00)	0.00 (0.00,13.50)	13.00 (0.00,27.00)	8.00 (0.00,13.25)	19.00 (15.50,29.00)	12.50 (6.00,23.25)	χ^2^ = 13.52#	0.009
GFOG, M (Q₁, Q₃)	17.80 (15.95, 19.22)	18.55 (16.15,20.40)	17.30 (15.25,18.97)	18.00 (17.38,19.32)	17.20 (14.00,18.25)	17.50 (16.30,19.28)	χ^2^ = 5.26#	0.262
FKGL, M (Q₁, Q₃)	16.27 (14.08, 18.16)	17.83 (15.79,19.23)	15.62 (14.00,18.85)	17.16 (16.33,18.64)	14.19 (13.54,16.03)	15.86 (14.08,16.62)	χ^2^ = 14.71#	0.005
CL, M (Q₁, Q₃)	18.89 (16.46, 20.49)	20.59 (17.24,22.29)	16.87 (13.98,19.44)	18.97 (18.02,20.57)	17.54 (16.40,19.06)	19.18 (17.12,20.02)	χ^2^ = 9.16#	0.057
SMOG, M (Q₁, Q₃)	13.91 (12.12, 15.23)	14.21 (12.77,15.86)	13.91 (11.75,15.27)	14.40 (14.05,15.33)	12.36 (11.48,14.02)	13.27 (12.55,14.40)	χ^2^ = 11.01#	0.026
LW, M (Q₁, Q₃)	54.00 (51.00, 58.00)	50.00 (48.75,56.25)	55.00 (49.50,58.00)	54.00 (51.75,59.00)	54.50 (52.75,58.00)	56.00 (51.75,58.25)	χ^2^ = 6.86#	0.144

F: ANOVA, #: Kruskal-Wallis test. SD: standard deviation, M: Median, Q: 1st Quartile, Q₃: 3st Quartile.

### Clinical intent alignment (CIA) analysis

Clinical Intent Alignment (CIA) analysis was conducted to assess the extent to which LLM-generated responses covered guideline-defined medical key points derived from the 2025 EASL Clinical Practice Guidelines. As shown in Table 2, CIA scores were generally comparable across the five evaluated models, with median values ranging from 0.65 to 0.70. Statistical comparison using the Kruskal–Wallis test revealed no significant difference among models (χ^2^ = 1.60, *p* = 0.809), indicating that the evaluated LLMs demonstrated broadly similar performance in covering core clinical intent. At the content level (Table 3), CIA scores differed significantly across AIH health education themes (χ^2^ = 17.96, *p* = 0.001). The highest CIA scores were observed for etiopathogenesis-related questions (median 0.80), whereas treatment and management topics showed comparatively lower alignment (median 0.60). Other themes, including clinical manifestations, diagnostic examinations, and prognosis and follow-up, demonstrated intermediate levels of guideline alignment.

### Quality analysis

**Figure 1:**
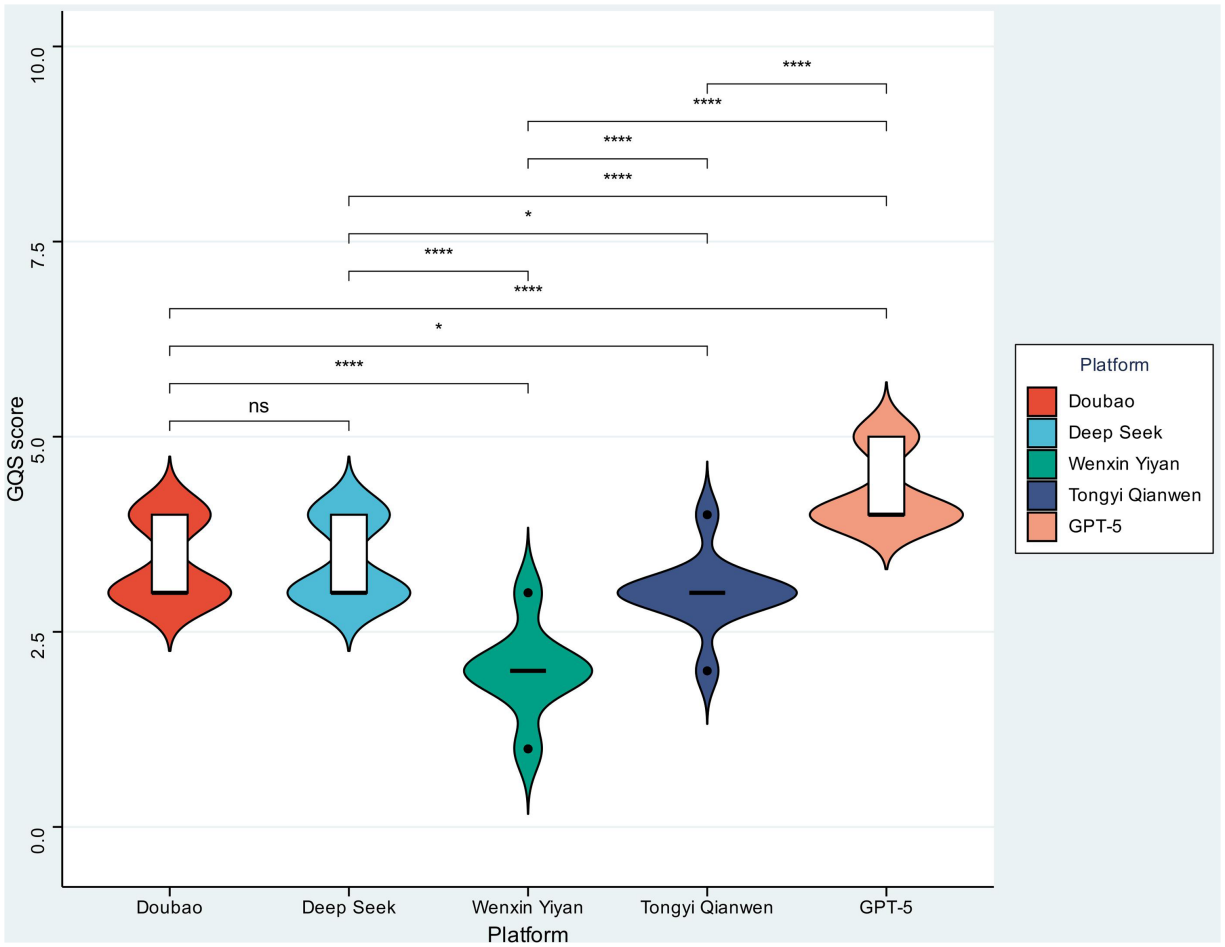
Comparison of GQS scores across five mainstream large language models. Violin plots show the distribution of GQS scores for Doubao, Deep Seek, Wenxin Yiyan, Tongyi Qianwen, and GPT-5. The embedded boxplots indicate the median and interquartile range. Pairwise comparisons between platforms are shown above the plots, with significance levels indicated by asterisks and “ns” denoting non-significant differences.

As shown in Figure 1, the distribution of GQS scores demonstrated clear differences in overall text quality among AI platforms. ChatGPT achieved the highest GQS scores, with a median value approaching the upper limit of the scale (5.0) and the narrowest interquartile range (4.9–5.0). The highly concentrated violin plot distribution indicates strong consistency and stability in the quality of texts generated by this model. Doubao (median: 5.0; IQR: 4.8–5.0) and DeepSeek (median: 5.0; IQR: 4.7–5.0) also showed relatively concentrated score distributions, with no statistically significant difference between the two platforms (*p* > 0.05). Compared with ChatGPT, their slightly wider interquartile ranges suggest marginally lower consistency.

In contrast, Wenxin Yiyan (median: 3.5; IQR: 2.5–4.2) and Tongyi Qianwen (median: 2.5; IQR: 1.8–3.5) exhibited substantially lower GQS scores. One-way ANOVA confirmed significant differences in GQS scores across platforms (*p* < 0.001). According to standard GQS interpretation, scores of 4–5 indicate high-quality information, whereas a score of 3 represents moderate quality. The corresponding violin plots showed broader distributions with pronounced long-tail characteristics, indicating greater variability in text quality and a higher proportion of low-quality outputs.

**Figure 2:**
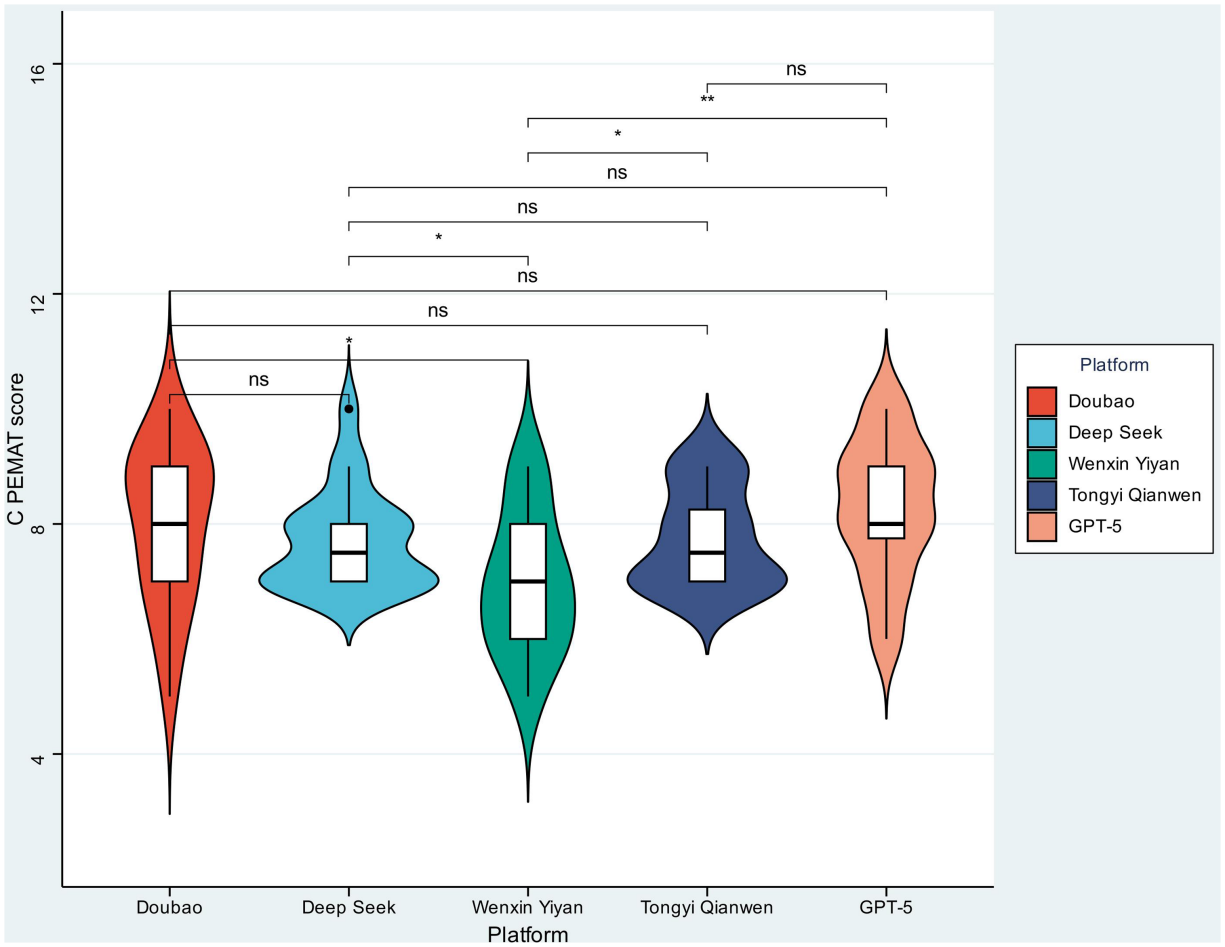
Comparison of C-PEMAT scores across five mainstream large language models. Violin plots show the distribution of C-PEMAT scores for Doubao, Deep Seek, Wenxin Yiyan, Tongyi Qianwen, and GPT-5. The embedded boxplots indicate the median and interquartile range. Pairwise comparisons between platforms are shown above the plots, with significance levels indicated by asterisks and “ns” denoting non-significant differences.

As illustrated in Figure 2, C-PEMAT scores across platforms were mostly between 7 and 8, indicating generally good understandability and actionability of the generated patient education materials. Pairwise comparisons indicated that most inter-platform differences were not statistically significant, although significant differences were observed between specific platform pairs (*p* < 0.05). Notably, ChatGPT and Doubao exhibited relatively higher median scores and narrower interquartile ranges, reflecting more stable performance in generating patient-appropriate educational content. In contrast, Wenxin Yiyan showed a more dispersed score distribution, indicating greater variability in educational suitability. DeepSeek and Tongyi Qianwen demonstrated intermediate performance, with substantial overlap in score distributions relative to other models.

Overall, while differences in C-PEMAT scores among platforms were modest, certain models showed advantages in generating content more aligned with patient education suitability criteria, suggesting that the generated materials may be broadly acceptable for patient-oriented health education.

### Correlation analysis

**Figure 3:**
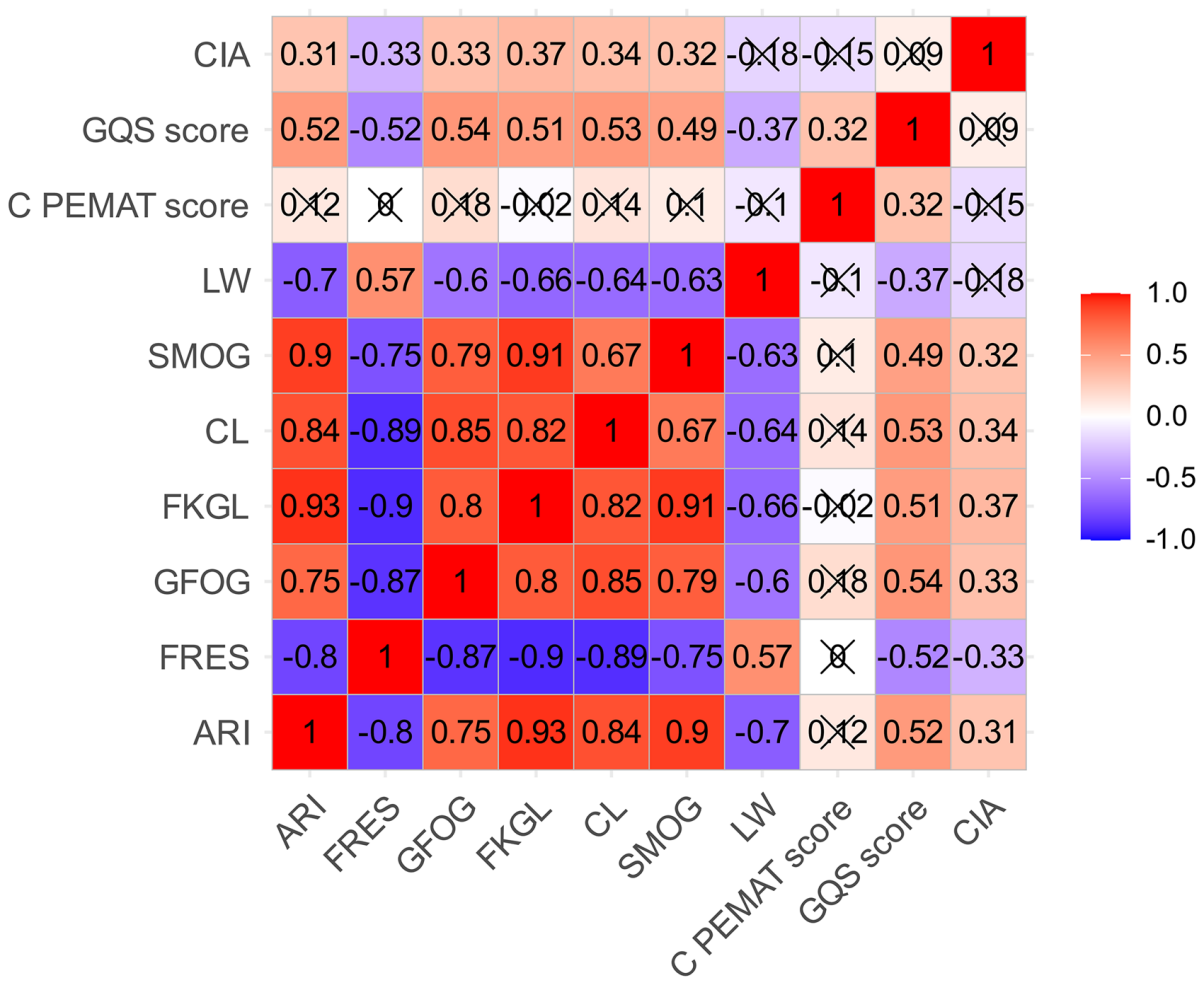
Correlation heatmap of readability and quality assessment metrics. The heatmap shows correlation coefficients among readability indices and quality assessment metrics, including ARI, FRES, GFOG, FKGL, CL, SMOG, LW, C-PEMAT score, GQS score, and CIA. Red indicates positive correlation and blue indicates negative correlation. Black crosses mark non-significant correlations.

As shown in Figure 3, correlations between quality metrics and readability indices were generally weak to moderate, suggesting that linguistic complexity and content quality represent related but distinct dimensions in AI-generated AIH patient education materials. Nonetheless, several meaningful correlation patterns were identified.

In the quality dimension, GQS demonstrated moderate positive correlations with multiple grade-level readability indices, including ARI (*r* = 0.52), GFOG (*r* = 0.54), FKGL (*r* = 0.51), CL (*r* = 0.53), and SMOG (*r* = 0.49), as well as a moderate negative correlation with FRES (*r* = −0.52). These findings indicate that texts receiving higher quality ratings tended to exhibit more complex syntactic structures and vocabulary usage. In addition, GQS showed a positive correlation with LW (*r* = 0.32), suggesting that greater content elaboration or information density may contribute to higher perceived quality.

In contrast, C-PEMAT scores exhibited minimal associations with traditional readability indices. Correlation coefficients between C-PEMAT and ARI, FRES, GFOG, FKGL, CL, and SMOG were close to zero, indicating that patient education suitability was largely independent of sentence complexity and vocabulary difficulty. Only very weak correlations were observed with LW (*r* ≈ −0.10) and SMOG (*r* ≈ −0.15). The slight negative association with SMOG suggests that excessive lexical complexity may marginally reduce text understandability and actionability, although the effect size was limited.

Similarly, Clinical Intent Alignment (CIA) scores showed only weak correlations with both readability indices and quality metrics. CIA demonstrated a modest positive association with grade-level readability indices such as ARI (*r* = 0.31), GFOG (*r* = 0.33), FKGL (*r* = 0.37), CL (*r* = 0.34), and SMOG (*r* = 0.32), as well as a weak negative correlation with FRES (*r* = −0.33). In contrast, correlations between CIA and C-PEMAT (*r* ≈ −0.15) and GQS (*r* ≈ 0.09) were minimal. These results suggest that guideline-aligned clinical content coverage represents a relatively independent evaluation dimension that is not strongly determined by linguistic complexity or perceived information quality.

Further examination of the internal structure of readability metrics revealed strong and consistent correlations among grade-level indices. Significant positive correlations were observed between ARI and FKGL (*r* = 0.93), FKGL and SMOG (*r* = 0.91), and ARI and SMOG (*r* = 0.90). In contrast, FRES showed strong negative correlations with these indices, such as FKGL (*r* = −0.90) and GFOG (*r* = −0.87). These findings indicate strong consistency among readability formulas in assessing reading difficulty, although their explanatory value for overall information quality and patient education suitability appears limited.

## Discussion

In this study, we evaluated LLM-generated patient education texts for autoimmune hepatitis (AIH) from two complementary perspectives: model type and health education content category. Using a multidimensional framework incorporating information quality (GQS), patient education suitability (C-PEMAT), clinical intent alignment (CIA), and multiple readability indices, we examined the relationships among text quality, guideline-aligned clinical content coverage, and linguistic complexity. Our findings indicate that model type is the primary determinant of patient education text quality, whereas content category mainly influences linguistic complexity. Overall, text quality, clinical intent alignment, and readability appear to represent related but largely independent evaluation dimensions. From a clinical perspective, most models generated information of moderate-to-good quality, although relatively high readability levels suggest that further simplification may be needed for general patient populations.

Previous studies have examined the performance of LLMs in medical information dissemination across various clinical domains. For example, Duran et al. evaluated the readability, clarity, and accuracy of responses generated by multiple LLMs, including ChatGPT-4, Gemini, and Copilot, in the field of plastic surgery, and found that ChatGPT-4 performed more consistently in producing patient-oriented, accurate, and comprehensive medical information (22). Consistent with these findings, our results demonstrated significant differences among LLMs in both GQS and C-PEMAT scores. ChatGPT achieved the highest performance across both core metrics, with highly concentrated score distributions, suggesting strong consistency under the conditions of this study. In contrast, certain models, such as Wenxin Yiyan and Tongyi Qianwen, exhibited not only lower overall quality scores but also greater score dispersion, indicating uncertainty in content completeness, logical coherence, and informational consistency. Similar inter-model variability in AI-generated health education materials has been reported in other disease contexts (23). These findings underscore that model selection itself may serve as a critical determinant of information quality when LLM-generated content is applied in clinical or patient education settings.

From a mechanistic perspective, inter-model differences may reflect variations in medical knowledge coverage within training corpora, the proportion of health education–oriented texts, and the emphasis on patient-friendly language during instruction tuning. Models more extensively exposed to patient guidelines, health-related Q&A datasets, and evidence-based medical summaries may generate outputs with greater informational completeness and clearer structure (24–26). Some models may also be optimized for simplifying medical terminology and providing actionable recommendations, thereby improving performance in C-PEMAT domains (27). Nevertheless, these explanations remain speculative and warrant further investigation across multiple technical dimensions. Beyond differences in training corpora composition, inter-model variability may also relate to architectural heterogeneity—such as dense transformer structures versus mixture-of-experts (MoE) designs—which differ in parameter activation strategies and computational specialization (28). Furthermore, variations in post-training alignment approaches, particularly the relative depth of supervised fine-tuning (SFT) and reinforcement learning from human feedback (RLHF), may shape response style, safety constraints, and adherence to guideline-consistent recommendations (29). Recent reasoning-oriented configurations, such as structured “thinking” modes or Chain-of-Thought prompting, may enhance logical coherence and reduce superficially plausible yet clinically inappropriate suggestions (30). However, reasoning transparency does not necessarily guarantee factual accuracy or clinical safety (31). Collectively, architectural design, alignment optimization, and reasoning strategies likely interact to produce the inter-model variability observed in this study.

With respect to readability, significant differences were observed among LLMs across multiple indices, including ARI, FRES, GFOG, FKGL, CL, SMOG, and LW, reflecting systematic variation in syntactic organization and lexical selection strategies. In patient education contexts involving complex chronic diseases such as AIH, these differences have important practical implications. Excessive linguistic complexity may hinder patient comprehension, whereas excessive simplification may compromise informational accuracy. Therefore, the suitability of LLMs for health education should be evaluated through balanced consideration of both readability and content quality.

At the content level, different AIH health education themes exhibited systematic differences in readability. Texts related to treatment management and follow-up achieved higher C-PEMAT scores overall, whereas content focusing on disease etiology, pathogenesis, and diagnostic evaluation required higher reading grade levels. Previous studies have similarly shown that medical texts addressing disease mechanisms and diagnostic criteria tend to demonstrate higher linguistic complexity, including AI-generated patient education materials. For instance, educational content related to neuro-oncology or oncologic diseases often exceeds recommended readability levels (32). In contrast, guidance-oriented content focusing on patient management and follow-up is more amenable to structured presentation and linguistic simplification, facilitating improved readability and comprehension (33). Notably, GQS scores did not differ significantly across content themes in our study, suggesting that among higher-performing models, overall information quality can remain relatively stable despite variations in thematic complexity. This finding has practical implications for clinical application, indicating that with appropriate model selection, diverse educational topics may be delivered without substantial compromise in overall quality.

Correlation analysis further demonstrated moderate positive associations between GQS and grade-level readability indices such as ARI, FKGL, and SMOG, alongside a negative correlation with FRES. These findings suggest that high-quality patient education texts are often characterized by higher information density and a certain degree of professional expression, rather than extreme linguistic simplification (34). In contrast, patient education suitability as measured by C-PEMAT showed generally weak correlations with traditional readability metrics, with only very weak associations observed for SMOG and LW. This pattern aligns with previous research. Lipari et al. reported no significant correlation between PEMAT scores and readability indices in online diabetes education materials (35), and similarly weak associations were observed in assessments of patient education materials for vocal fold paralysis (36). These findings suggest that patient education suitability is influenced more by information organization, logical structure, and action-oriented design than by sentence length or vocabulary complexity.

In addition, strong correlations among readability indices were observed, confirming their consistency and stability in measuring linguistic complexity. However, their limited explanatory power with respect to text quality and patient education suitability further supports the necessity of adopting a multidimensional evaluation framework for AI-generated medical texts, rather than relying on a single readability indicator (37).

Several limitations of this study should be acknowledged. First, the sample size was relatively limited, including only five LLMs and five health education content categories, which may not fully capture variability across different model architectures or disease contexts. Although the question framework was informed by the 2025 EASL Clinical Practice Guidelines, certain specialized clinical scenarios were not separately evaluated, which may limit comprehensive guideline representation. Second, readability was assessed using English-translated versions of originally Chinese-generated responses; thus, findings should be interpreted as relative indicators of textual complexity rather than precise measures of native-language readability. Third, the study relied primarily on expert-based evaluation tools and did not incorporate patient involvement in either question development or response evaluation. Consequently, the assessment was based on objective instruments rather than direct patient-reported comprehension, which may limit insight into real-world usability. Finally, individual patient characteristics such as age, educational background, and health literacy were not stratified, which may constrain the generalizability of findings in the context of precision health communication. In addition, multiple statistical comparisons were conducted without formal correction, which may increase the risk of type I error and therefore the results should be interpreted with caution.

Future research could expand the range of evaluated models and health education topics, particularly in specialized and high-risk clinical scenarios such as pregnancy management or autoimmune overlap syndromes. In addition, incorporating patient-centered evaluation approaches—such as patient comprehension testing or user-experience studies—would provide more direct evidence of the real-world usability of AI-generated health education materials. Developing integrated evaluation frameworks that combine objective text metrics, user experience, and clinical behavioral outcomes (e.g., medication adherence or follow-up compliance) may further improve the assessment of AI-generated medical information. Finally, leveraging the controllability of LLMs to tailor linguistic complexity and information structure to individual health literacy levels may facilitate more personalized and sustainable health information dissemination.

## Conclusion

This study systematically evaluated AI-generated patient education texts for autoimmune hepatitis by integrating model type and health education content category as key analytical factors, using patient education suitability (C-PEMAT), overall information quality (GQS), clinical intent alignment (CIA), and multiple readability indices. The findings demonstrate that model type is the primary determinant of overall text quality and educational suitability, whereas content category mainly influences linguistic complexity and reading difficulty.

Overall, most evaluated models generated patient education content of moderate-to-good quality and demonstrated comparable alignment with guideline-defined clinical intent. However, the relatively high readability levels observed in several models suggest that further simplification may be necessary to ensure accessibility for general patient populations.

Further correlation analyses indicate that text quality, guideline-aligned clinical content coverage, and readability represent related but largely independent evaluation dimensions. While moderate information depth and appropriate use of professional terminology may enhance overall quality, excessive linguistic simplification may undermine the accuracy and completeness of medical information.

Based on these findings, we recommend prioritizing LLMs with stable performance in information quality and patient education suitability when implementing AI-assisted health communication strategies. At the same time, readability should be selectively optimized according to topic complexity and the health literacy level of the target population to enhance the scientific rigor and practical value of AI-supported patient education.

## Data Availability

The raw data supporting the conclusions of this article will be made available by the authors, without undue reservation.

## Footnotes

1
https://chatgpt.com

2
https://www.doubao.com/chat/

3
https://chat.deepseek.com

4
https://yiyan.baidu.com

5
https://www.tongyi.com

6
http://readabilityformulas.com
