# Supplementary material for: Performance evaluation of mainstream large language models in autoimmune hepatitis patient education: a comparative study of readability, quality, and reliability
Source: Front Public Health. 2026 Mar 20;14:1805848. doi: 10.3389/fpubh.2026.1805848 (PMC13047142; doi:10.3389/fpubh.2026.1805848)
Supplement: Supplementary file 1 [file Data_Sheet_1.docx]

Supplementary Table S1：Full Prompt Disclosure and Interaction Protocol

| **Prompting Strategy** |
| --- |
| The study adopted a zero-shot prompting strategy. The 20 predefined questions listed in Table 1 were directly entered into each large language model via the public web interface.No additional system-level instructions, role assignments (e.g., “You are a hepatology expert”), formatting constraints, or chain-of-thought guidance were provided.No health literacy framing (e.g., “explain to a 5th grader” or “explain to a medical student”) was applied. |
| **Model Configuration** |
| All models were accessed through their default public web interfaces.Default generation parameters were maintained across platforms. No manual adjustments were made to temperature, maximum token length, or reasoning settings beyond the platform’s default configuration.For DeepSeek, the “deep thinking mode” was enabled as provided by the web interface default setting at the time of testing. |
| **Example Interaction** |
| User input: “What is the core immune abnormality causing autoimmune hepatitis?”  Model output: The core immune abnormality in autoimmune hepatitis (AIH) is a breakdown of immune tolerance to hepatocyte-specific antigens, leading to uncontrolled autoreactive T-cell activation and B-cell production of autoantibodies. This results in a persistent inflammatory response targeting hepatocytes, driven by dysregulation of regulatory T cells (Tregs) and overexpression of pro-inflammatory cytokines (e.g., IFN-γ, TNF-α). The immune system fails to distinguish self from non-self, causing chronic hepatocellular damage and progressive fibrosis. Genetic predisposition, environmental triggers, and epigenetic factors synergize to initiate this pathogenic cascade, with loss of central and peripheral tolerance as the pivotal event. |
